# Rhabdomyolysis in Children: A State-of-the-Art Review

**DOI:** 10.3390/children12040492

**Published:** 2025-04-10

**Authors:** Manson Chon In Kuok, Winnie Kwai Yu Chan

**Affiliations:** Department of Paediatrics, Queen Elizabeth Hospital, Hong Kong, China; chankyw@ha.org.hk

**Keywords:** rhabdomyolysis, myositis, creatine kinase, acute kidney injury

## Abstract

Rhabdomyolysis in pediatric patients is a rare but potentially life-threatening condition characterized by the breakdown of skeletal muscle fibers, leading to the release of intracellular components such as myoglobin, potassium, and creatine kinase into the bloodstream. This process can result in severe electrolyte imbalances and acute kidney injury (AKI), sometimes necessitating kidney replacement therapy. While rhabdomyolysis is well studied in adults, pediatric cases present unique diagnostic and therapeutic challenges due to distinct etiologies and clinical manifestations. This review explores the pathophysiology, etiologies, complications, treatment, and outcomes of rhabdomyolysis, with a particular focus on the pediatric population. Emerging evidence regarding the role of hemoadsorption in myoglobin removal is discussed and summarized. Additionally, we propose a systematic framework for the management and monitoring of these patients.

## 1. Introduction

Rhabdomyolysis refers to the breakdown of skeletal muscle fibers, resulting in the release of intracellular components, including electrolytes, myoglobin, and creatine kinase, into the bloodstream. While some cases resolve spontaneously with no complications, others can be complicated with significant electrolyte disturbances and acute kidney injury (AKI) requiring kidney replacement therapy (KRT).

The association between muscle injury and renal failure was first identified back in the 1940s [1]. Since then, advances in understanding its pathophysiology and management have emerged in the past decades. While rhabdomyolysis is more well studied in adults, pediatric cases have unique diagnostic and therapeutic challenges due to distinct etiologies and clinical presentations.

Early recognition and timely interventions are the keys to prevent or mitigate the progression of AKI. This review aims to provide an updated overview on the current understanding, evaluation, and management of rhabdomyolysis with a focus on the pediatric population. To include the latest research on this topic, a PubMed search was conducted in Clinical Queries in December 2024 using the keywords “rhabdomyolysis” and “crush syndrome”, limited to studies and reviews published between 2020 and 2024 for patients aged from birth to 18 years old.

## 2. Definitions

There have been variations in the diagnostic criteria for rhabdomyolysis across studies [2]. Most commonly, the condition is defined by elevated serum creatine kinase (CK) or myoglobin levels, as these biomarkers are released from damaged muscle tissues and reflect the extent of muscle injury. The presence of excessive myoglobin is pathognomonic of rhabdomyolysis; however, quantitative measurement of serum myoglobin may not be available in many healthcare settings. In addition, myoglobin has a relatively short half-life of 1 to 3 h, making it a less sensitive marker with possibility of false-negative results [3].

A recent systematic review recommended defining rhabdomyolysis as elevated CK > 1000 IU/L, or exceeding five times the upper limit of normal for the local laboratory, accompanied by muscle-related symptoms [4].

## 3. Symptoms

The classical triad of rhabdomyolysis includes myalgia, muscle weakness, and tea-colored urine. However, this full triad is observed in only less than 10% of patients [5,6]. The location of muscle pain and weakness depends on the site of injury, with weakness most commonly affecting the thighs, and pain frequently localized to the thighs and calves.

The change in urine color is due to the presence of myoglobin, and is described by patients and parents as dark, tea-colored, or cola-colored. Myoglobin released into the circulation following muscle injury is filtered by the kidneys and enters the urine when plasma myoglobin levels exceed the renal threshold of 0.5 to 1.5 mg/dL. The severity of myoglobinuria depends on circulating myoglobin levels, glomerular filtration rate, and urine flow. Gross myoglobinuria leading to visible urine discoloration typically occurs when serum myoglobin exceeds 100 mg/dL [7]. Urine myoglobin can produce a false-positive result for “blood” on urine dipstick testing due to its heme-containing nature, but it can be differentiated from true hematuria by the absence of red blood cells under microscopy. Some laboratories offer quantitative urine myoglobin measurement for confirmation.

## 4. Epidemiology

In adults, approximately 26,000 cases of rhabdomyolysis were reported annually in the United States during the 1990s [8]. More recent data from the US inpatient database indicate an average of 37,000 rhabdomyolysis-related hospitalizations per year between 2016 and 2018 [9]. A similar upward trend has also been observed in the pediatric population, with a review showing a three-fold increase in the rate of rhabdomyolysis-related pediatric hospitalizations, rising from 0.8 to 2.8 per 100,000 children from 2006 to 2016 [10]. It is difficult to determine whether the incidence of rhabdomyolysis is genuinely increasing or if the rise is due to greater awareness of the condition. Estimating the global burden of rhabdomyolysis remains challenging, as some contributing factors, such as the prevalence of viral infections, climate conditions (temperature and humidity) that predispose to exertional rhabdomyolysis, and the frequency of natural disasters, can vary significantly across countries. Furthermore, mild cases of rhabdomyolysis may be overlooked, as these patients can recover spontaneously.

## 5. Pathophysiology

### 5.1. Rhabdomyolysis

The mechanisms of rhabdomyolysis are primarily related to either direct muscle trauma with rupture of the plasma membrane or adenosine triphosphate (ATP) depletion within myocytes [2]. Normally, myocytes have lower intracellular calcium levels, and this electrolyte homeostasis is maintained by energy-dependent ion pumps, which are crucial in maintaining cellular integrity. Dysfunction of these pumps, as a result of direct injury or ATP depletion, leads to excessive intracellular calcium influx, which in turn (i) leads to sustained myofiber contraction, which further depletes cellular energy reserves; (ii) activates calcium-dependent proteases and phospholipase, resulting in the degradation of structural components of cellular and mitochondrial membranes; (iii) releases cytotoxic intracellular components and produces reactive oxygen species (ROS), which further accentuate the inflammatory and necrotic process, leading to cell death. Reperfusion injury to the ischemic skeletal muscle and fluid sequestration can further cause myocyte swelling and loss of cellular integrity, resulting in cell death and cascades of intracellular contents release and ROS production.

### 5.2. Acute Kidney Injury

**Figure 1:**
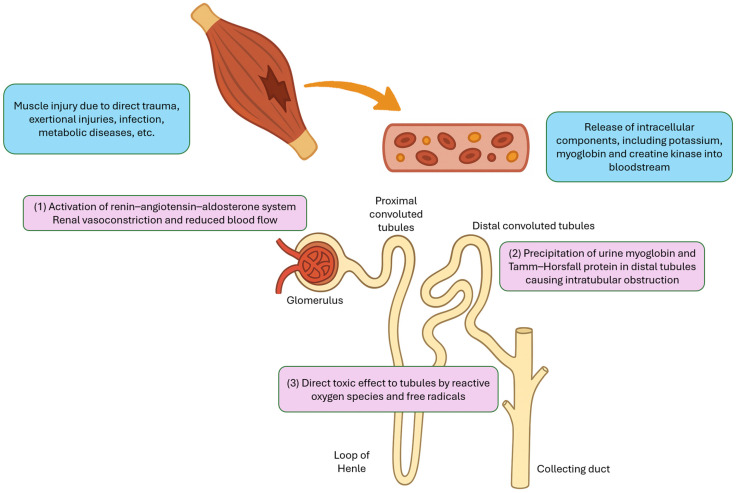
Major mechanisms of acute kidney injury in rhabdomyolysis.

The pathophysiology of rhabdomyolysis-induced AKI is multifactorial and can generally be categorized into three major mechanisms (Figure 1). First, fluid sequestration in damaged muscle tissue leads to secondary activation of the renin–angiotensin–aldosterone system, which causes renal vasoconstriction and reduced renal blood flow. Second, urine myoglobin can interact with Tamm–Horsfall protein, particularly in acidic environment, resulting in precipitation and cast formation in the distal tubules, which leads to intratubular obstruction. Third, the reactive oxygen species and free radicals released from damaged cells can exert direct toxic effects on the renal tubules [7].

## 6. Etiologies

The etiologies of rhabdomyolysis can broadly be classified into three categories: (i) direct muscle trauma, (ii) exertional injury leading to energy depletion, and (iii) non-traumatic, non-exertional causes (Table 1).

### 6.1. Direct Muscle Trauma

Rhabdomyolysis due to physical trauma, also known as crush syndrome, can occur in children due to natural disasters [11,12] and other pediatric trauma such as road traffic accidents and falls [13,14,15]. Extremity injuries can lead to muscle edema and compartment syndrome, which further exacerbate tissue necrosis [16]. Other causes of direct muscle injuries include electrical shock, burns, and heat strokes [17,18,19]. Traumatic rhabdomyolysis following physical child abuses have also been reported [20,21,22].

### 6.2. Exertional Injuries

Rhabdomyolysis can arise when muscle energy demand exceeds the energy supply, such as after strenuous physical exertion. It has been reported in conditions such as athletic training and competitions, as well as corporal punishment involving repetitive exercises [23,24,25]. Additionally, patients with hyperkinetic states, for example dystonia or status epilepticus, may also develop rhabdomyolysis.

Malignant hyperthermia (MH) and neuroleptic malignant syndrome (NMS) are two clinical syndromes with overlapping features, including hyperthermia, muscle rigidity and rhabdomyolysis, yet distinct pathogenesis and triggering factors. MH is characterized by a hypermetabolic response to depolarizing muscle relaxants or volatile anesthetic agents like sevoflurane and halothane [26]. The condition predominantly affects individuals with an inherited mutation in the ryanodine receptor gene (RYR-1), which results in a defective calcium channel in the sarcoplasmic reticulum of skeletal muscle. This defect leads to unregulated calcium release into the intracellular space, causing sustained muscle rigidity and damage. On the other hand, NMS is hypothesized to arise from a central dopamine receptor blockade in the hypothalamus, leading to dysregulation of thermoregulatory pathways and muscle tone, typically triggered by antipsychotic medications [27,28].

In addition, serotonin syndrome associated with anti-depressants [29,30], alongside alcohol intoxication [31] and various recreational drugs such as heroin, ecstasy, cannabis, amphetamine, and cocaine [32,33], are other important causes of rhabdomyolysis, especially in adolescents. A term “Raver’s hematuria” has been used to describe the phenomenon of rhabdomyolysis following the use of recreational drugs and all-night dancing at rave parties [34].

### 6.3. Non-Traumatic and Non-Exertional

#### 6.3.1. Infection

Viral myositis is the most common causes of rhabdomyolysis in children. The course is mostly benign and self-limiting with low risk for AKI, and hence the term “benign acute childhood myositis” [35]. Affected children, predominantly aged 5 to 9 years old [36], often present with calf tenderness, gait disturbances, and refusal to walk in the context of upper respiratory tract infection. Influenza viruses, particularly Influenza B, are the commonest pathogens [37,38,39]. Direct viral invasion, viral toxins, and cytokine-mediated muscle damage are the postulated mechanisms [40]. Other reported viral agents include parainfluenza, enterovirus, herpes simplex, varicella zoster virus, and Dengue fever [7,41]. In recent years, there were also reports of rhabdomyolysis associated with COVID-19 infection [42,43,44] and multi-system inflammatory syndrome in children (MIS-C) [45,46]. A post-infectious complication typically occurs 2 to 6 weeks after COVID-19 infection.

Bacterial infections, although less common than viral myositis, have also been documented as a cause of rhabdomyolysis. These included Streptococcus pyogenes, Staphylococcus aureus, and Salmonella species infections [7].

#### 6.3.2. Metabolic

Pediatric studies showed that 3% to 5% of rhabdomyolysis were attributed to underlying metabolic disorders [13,14,47]. These conditions primarily affect energy production and muscle metabolism, leading to ATP deficit and subsequent muscle damage. These patients often experience muscle symptoms in all skeletal muscles, which include neck, jaw, arm, and paraspinal muscles [48]. The common inherited disorders associated with rhabdomyolysis include glycogen metabolism disorders, fatty acid metabolism disorders, and mitochondrial diseases.

Rhabdomyolysis due to glycogen metabolism disorders is characterized by symptoms triggered by brief isometric exercises (e.g., heavy lifting) or sustained dynamic exercise (e.g., climbing stairs, walking uphill), with symptom onset typically within minutes of exertion. Common causes include McArdle’s disease (glycogen storage disease type V with phosphorylase deficiency) and Tarui’s disease (glycogen storage disease type VII with phosphofructokinase deficiency).

Long-chain fatty acid is a major energy source for muscles during prolonged exercise. Therefore, rhabdomyolysis related to underlying lipid metabolism disorders is typically triggered by prolonged aerobic exercises and can also be exacerbated by fasting or febrile illnesses. Common examples include carnitine palmitoyltransferase II (CPT II) deficiency and various types of acyl-coenzyme A dehydrogenase deficiency.

Mitochondria are intracellular organelles responsible for ATP generation, and skeletal muscles are particularly susceptible to mitochondrial dysfunction due to their high energy requirements. Muscle symptoms are usually progressive, but some patients may also present acutely with rhabdomyolysis [49,50].

A detailed discussion of the various types of metabolic myopathies is beyond the scope of this review. Further readings of review articles by Darras et al. are recommended for more in-depth discussions [51,52]. The key message is to maintain awareness of potential underlying metabolic conditions in rhabdomyolysis patients, particularly in children with recurrent episodes, a family history of rhabdomyolysis, or rhabdomyolysis triggered by fasting.

#### 6.3.3. Drugs and Toxins

Statins are a well-known class of drug that can cause muscle symptoms and rhabdomyolysis. Though the incidence of statin-associated rhabdomyolysis is not particularly high (0.3–13.5 cases per 1,000,000 prescriptions) [53], the widespread use of these drugs, especially in adults, makes it important to recognize this complication. More than 150 drugs have been identified as potential triggering factors for rhabdomyolysis, and some commonly used medications in children and adolescents are summarized in Table 2 [41,54,55,56,57]. Additionally, intoxication due to bee stings, snake or insect bites were also reported [58,59,60].

#### 6.3.4. Other

Some other causes of rhabdomyolysis include electrolyte disturbances such as hypokalemia and hypophosphatemia [61,62,63], status asthmaticus [64,65], and endocrinopathies such as Hashimoto thyroiditis [66] and diabetic ketoacidosis [67,68].

##### Etiology: Children vs. Adults

The etiology of rhabdomyolysis differs between adults and children. In adults, sepsis and drug-related causes are the most common, whereas in the pediatric population, viral infections and trauma are the leading triggers. Among children, viral infections are more prevalent in those under 12 years old, while trauma is more commonly observed in older children and adolescent groups [5,38,47,69].

## 7. Complications

Early complications of rhabdomyolysis mainly involve electrolyte disturbances and their manifestations. Late complications, such as acute kidney injury and disseminated intravascular coagulopathy (DIC), typically arise 12 to 72 h following the initial insult [70].

### 7.1. Early Complications

#### 7.1.1. Hyperkalemia

Around 98% of potassium is present within the intracellular compartment [71], and muscle damage can lead to potassium release into the bloodstream, causing hyperkalemia. The electrolyte disturbance can further be exacerbated by co-existing renal dysfunction and metabolic acidosis. Uncontrolled hyperkalemia can potentially lead to fatal heart blocks and ventricular arrhythmias. As hyperkalemia often remains asymptomatic until cardiac toxicity develops, this highlights the importance of the close monitoring of electrolytes for early detection. Early ECG signs of hyperkalemia include tall T waves, the widening and flattening of P waves, and progressive QRS complex prolongation as serum potassium levels increase [72,73].

#### 7.1.2. Hyperphosphatemia, Early Hypocalcemia Followed by Hypercalcemia

Besides potassium, intracellular phosphate is also released in rhabdomyolysis causing hyperphosphatemia. Hypocalcemia is also a common complication affecting around 40% of patients with rhabdomyolysis [74], and it is attributed to calcium influx during muscle cell damage and the precipitation of calcium phosphate in myocytes. Hypocalcemia further worsen cardiotoxicity related to hyperkalemia.

However, during the recovery phase, calcium levels may rebound, potentially leading to hypercalcemia as previously deposited calcium is mobilized and phosphate levels normalize [75]. Secondary hyperparathyroidism due to hypocalcemia in the early phase and elevated calcitriol level may also play a role in late-onset hypercalcemia [76].

#### 7.1.3. Metabolic Acidosis

High-anion-gap metabolic acidosis in rhabdomyolysis results from production of lactic acid secondary to muscle ischemia and the release of other organic acids from disrupted muscle cells. Metabolic acidosis can exacerbate hyperkalemia and has been demonstrated to be associated with the development of AKI in rhabdomyolysis in multiple pediatric studies [38,77,78].

### 7.2. Late Complications

#### 7.2.1. Acute Kidney Injury

While some cases of AKI can present earlier, particularly in patients with sepsis, others may manifest later, typically one to two days after muscle injury, as seen in exertional rhabdomyolysis [70,79]. Recent pediatric studies (2020–2024) reported a wide prevalence of AKI in pediatric rhabdomyolysis, ranging from 7% to 45% [10,14,38,69,80,81]. This variability likely reflects differences in study populations, study settings (e.g., emergency department vs. PICU), and AKI diagnostic criteria. Among different etiologies, patients with high-impact trauma, convulsions, and dystonia have a higher likelihood of developing AKI, whereas AKI is relatively uncommon in viral infections [19,38]. Other risk factors for AKI in pediatric rhabdomyolysis include higher peak CK levels [14,38,69,77,82], higher serum or urine myoglobin levels [19,69,77,81], metabolic acidosis or lower bicarbonate levels [38,77,78], abnormal urinalysis findings [14,38,47,69,83], and lower serum calcium levels [69,78]. In addition, severe rhabdomyolysis is often accompanied by other factors, such as dehydration, nephrotoxic medications, and sepsis, which further worsen the severity of AKI. Dialysis was required in 20% to 67% of pediatric patients with rhabdomyolysis-associated AKI [14,19,38,69,77,78,83].

#### 7.2.2. Disseminated Intravascular Coagulopathy

Different prothrombotic substances including thromboplastin are released during muscle necrosis, which activates coagulation cascade and subsequent disseminated intravascular coagulopathy (DIC). The prevalence of DIC in pediatric rhabdomyolysis ranged from 5% to 15% in different studies [69,84] and is associated with an increased risk of AKI. A meta-analysis in adults showed a pooled prevalence of DIC of 8.3% following rhabdomyolysis [85].

#### 7.2.3. Compartment Syndrome

Compartment syndrome is not uncommon, occurring in around 4% of severe rhabdomyolysis patients in adults [85]. Muscle damage can lead to fluid sequestration within myocytes, causing local edema and increased intramuscular pressure. Within confined spaces, especially in the extremities, this can compromise blood perfusion, resulting in ischemia and subsequently a self-perpetuating cycle of muscle damage. This risk can further be exacerbated with intravenous fluid during resuscitation. Clinical manifestations of compartment syndrome include diminished or absent peripheral pulses, pallor, pain, and sensory or motor deficits due to nerve damage.

## 8. Treatment and Prevention of AKI

**Figure 2:**
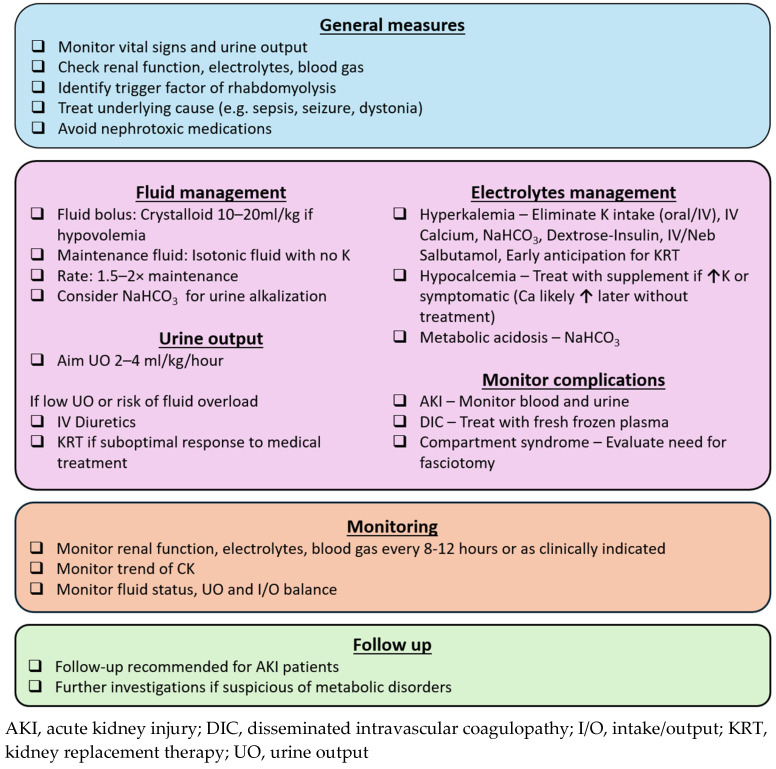
Approach to pediatric rhabdomyolysis.

The goals of rhabdomyolysis treatment include early recognition and correction of electrolyte disturbances, as well as measures to prevent and manage AKI. A practical approach is summarized in Figure 2.

### 8.1. General Measures

The underlying cause of rhabdomyolysis should be identified and treated to prevent ongoing muscle damage. This includes appropriate antibiotic therapy for sepsis, neurological medications for prolonged seizures and dystonia, and physical cooling for temperature dysregulation. Electrolyte abnormalities, particularly hyperkalemia, must be rapidly recognized and managed to minimize the risk of cardiac dysrhythmia. Since electrolytes and renal function can deteriorate with ongoing rhabdomyolysis, blood tests should be monitored every 8 to 12 h, or more frequently if clinically indicated in severe rhabdomyolysis. Additionally, nephrotoxic medications should be avoided to prevent further renal injury.

### 8.2. Fluid Replacement and Diuretics

Early and aggressive volume resuscitation is crucial in the management of rhabdomyolysis, as it enhances renal perfusion, dilutes nephrotoxic myoglobin, and promotes its elimination through urine. Initial fluid resuscitation with 10 to 20 mL/kg of crystalloid is recommended in cases of hypovolemic shock. However, to date, there were no large-scale randomized studies to establish the optimal fluid composition or infusion rate for maintenance fluids in children. A potassium-free isotonic solution is a reasonable initial choice, with close monitoring of electrolyte levels. Intravenous fluid administration at 1.5 to 2 times the maintenance rate is commonly advocated [41,86,87], targeting a urine output of 2 to 4 mL/kg/h, up to 200 to 300 mL/h in adults [7,41,87]. Close monitoring of blood pressure, and ideally central venous pressure is needed, particularly when administering high volumes of fluid.

Additionally, urine output should be carefully monitored, and diuretics should be considered if there is concern for fluid overload due to aggressive fluid resuscitation. Intravenous mannitol has been proposed for renal protection in rhabdomyolysis due to its theoretical benefits of renal vasodilatation and direct antioxidant effects on renal parenchyma [2,88], in addition to its diuretic properties. However, evidence remains inconclusive, especially in pediatric patients [3,41]. In cases of oliguric AKI not responding to diuretics, early anticipation of kidney replacement therapy is crucial, as fluid overload is strongly associated with increased morbidity and mortality [89].

### 8.3. Metabolic Acidosis and Urinary Alkalization

Urine alkalization with sodium bicarbonate, targeting a urine pH > 7, has been suggested in the treatment of rhabdomyolysis to mitigate AKI, primarily based on animal studies. Proposed mechanisms include the following: (i) reducing precipitation of myoglobin and Tamm–Horsfall protein, which is favored in acidic environment, and (ii) limiting redox (reduction–oxidation) cycling of myoglobin, lipid peroxidation and subsequent oxidative injury to renal tubules [7,88]. However, clinical studies have not consistently demonstrated superiority of bicarbonate over aggressive hydration alone [90,91]. Despite this, many authors still advocate for urine alkalization in both pediatric and adult centers, due to its relatively low risk and strong theoretical benefits. While further trials are needed to establish its efficacy, the use of sodium bicarbonate remains reasonable in patients with significant metabolic acidosis or concomitant hyperkalemia. However, its use is not without risks. Repeated administration can cause hypernatremia, and alkalization may reduce ionized calcium levels, potentially exacerbating the hypocalcemia commonly observed in the initial phase of rhabdomyolysis.

### 8.4. Kidney Replacement Therapy

Myoglobin, the primary nephrotoxin in rhabdomyolysis, has a relatively large molecular weight of 17,000 Daltons (for comparison: ammonia, 17 Daltons; urea, 60 Daltons; creatinine, 113 Daltons), making its clearance suboptimal with conventional extracorporeal approaches [92]. Currently, there is insufficient evidence to demonstrate a clear benefit of kidney replacement therapy (KRT) over conventional management in rhabdomyolysis and in preventing related AKI [93]. Therefore, the decision to initiate KRT should be guided by usual indications of KRT such as AKI with oliguria, fluid overload, severe and persistent hyperkalemia, or metabolic acidosis, rather than creatine kinase or myoglobin levels alone.

### 8.5. Hemoadsorption

Hemoadsorption for myoglobin removal has shown promising results over the past decade. Some hemoperfusion devices can adsorb hydrophobic molecules with molecular weights of up to 55 kDa, making them effective in removing myoglobin [94]. Additionally, the significantly larger surface area of these adsorption devices (45,000 m^2^) compared to standard KRT membranes (1.8 m^2^) further enhances their efficiency in myoglobin elimination [95]. Furthermore, these blood purification techniques facilitate the simultaneous removal of cytokines, which are well-known contributors to AKI in conditions such as sepsis-related rhabdomyolysis. A consensus statement for adults from the Hemoadsorption in Rhabdomyolysis Task Force recommends considering extracorporeal myoglobin removal via hemoadsorption if serum myoglobin levels exceed 10,000 ng/mL, with treatment ideally initiated within 24 h of admission [96]. Emerging case reports have also described the use of hemoadsorption in pediatric rhabdomyolysis patients [94,95,97,98,99] (Table 3). However, further studies are needed to determine the optimal timing for initiation and discontinuation, assess safety profiles in smaller children, and evaluate its effectiveness in kidney protection.

## 9. Prognosis and Outcomes

Mortality in pediatric rhabdomyolysis is rare in most studies. The reported incidence was 0.03% among hospitalized pediatric patients with rhabdomyolysis [10] but can rise to nearly 20% in studies conducted in PICU settings [13]. AKI appeared to be associated with mortality [13,69]; however, most death are attributed to underlying conditions such as sepsis and trauma rather than rhabdomyolysis itself [13,47].

Regarding long-term kidney outcomes, an adult study found that AKI severity and elevated serum myoglobin levels were associated with a decline in eGFR of more than 20 mL/min/1.73 m^2^ after three months [100]. Currently, data on the long-term outcomes of rhabdomyolysis in children remain scarce. One study reported that approximately 2% of affected patients developed renal complications, including proteinuria, hypertension, or a GFR of <90 mL/min/1.73 m^2^ [14]. Another study found that one in seven patients who required kidney replacement therapy (KRT) subsequently developed chronic kidney insufficiency [78]. Given an increased risk of developing chronic kidney disease following AKI in children, long-term follow-up in AKI patients is warranted for early detection and intervention [101,102].

## 10. Prevention

While physical trauma is difficult to predict, some causes of rhabdomyolysis are potentially preventable. Exertional rhabdomyolysis is especially relevant in the adolescent age group, and both patient and environmental factors can contribute to this condition. Individuals should be aware of their personal fitness levels, and preconditioning before engaging in strenuous activity is strongly recommended [103]. Additionally, exercising in high temperatures and humidity significantly increases the risk of exertional rhabdomyolysis. Wearing light-colored, loose-fitting breathable clothes can help heat dissipation, and maintaining adequate hydration is also crucial [104,105].

Patients with recurrent rhabdomyolysis should be evaluated for underlying metabolic disorders. Preventive measures such as avoiding prolonged exercise or fasting may help reduce the risk of recurrence. A detailed family history, especially any adverse reactions to anesthesia, may also uncover inherited conditions that predispose individuals to rhabdomyolysis triggered by general anesthetics [87].

Raising public awareness about rhabdomyolysis, including its warning symptoms, is essential. Prompt medical attention can facilitate early treatment and prevent severe complications such as acute kidney injury.

## 11. Conclusions

Pediatric rhabdomyolysis has diverse etiologies, and some cases may be complicated by life-threatening electrolyte disturbances and AKI. Early recognition, aggressive hydration, and prompt management of complications are crucial in reducing morbidity. Recently, hemoadsorption has shown promising results as an adjunct to conventional treatment. However, further research is needed to improve risk stratification and optimize treatment strategies.

## Figures and Tables

**Table 1:** Common causes of rhabdomyolysis.

Direct muscle trauma **Physical trauma** Electrical shock Burns Heat strokes
Exertional injury **Strenuous physical exertion, repetitive exercises** **Hyperkinetic states: dystonia, status epilepticus** Malignant hyperthermia Neuroleptic malignant syndrome Serotonin syndrome
Non-traumatic, Non-exertional **Infection** ▪ **Viral (most commonly Influenza B)** ▪ Bacterial **Underlying metabolic disorders** ▪ Glycogen metabolism disorders (e.g., McArdle’s disease, Tarui’s disease) ▪ Fatty acid metabolism disorders ▪ Mitochondrial diseases **Drugs (Table 2 in Section 6.3.3)** Toxins, bee stings, snake or insect bites Electrolyte disturbances: Hypokalemia, hypophosphatemia Status asthmaticus Endocrinopathy: Hashimoto thyroiditis, diabetic ketoacidosis

Common causes in children are in bold.

**Table 2:** Examples of drugs that can cause rhabdomyolysis.

Category	Drugs
Antimicrobials	Daptomycin, Macrolides (Azithromycin, Clarithromycin, Erythromycin), Meropenem, Trimethoprim-sulfamethoxazole, Linezolid, Piperacillin-tazobactam, Amphotericin B, Isoniazid
Neuropsychiatric medications	Lithium, Lamotrigine, Phenytoin, Phenobarbital, Selective Serotonin Reuptake Inhibitors (SSRIs), Antipsychotics (Quetiapine, Risperidone, Olanzapine)
Sedatives & anesthetics	Benzodiazepines, Barbiturates, Ketamine, Inhalation anesthetic, Chloral hydrate, Propofol, Succinylcholine
Miscellaneous	Statins, Steroids (Hydrocortisone, Dexamethasone, Mineralocorticoids), Acetaminophen, Salicylates, Theophylline

**Table 3:** Case reports of hemoadsorption in pediatric rhabdomyolysis.

Patient	Medical Condition	Laboratory Results	Outcomes
F/3	Altered sensorium	CK 349,600 IU/L	Substantial drop in CK, discharge from PICU on Day 10 [97]
M/4	Infantile epileptic encephalopathy with frequent seizure	CK 946,060 IU/L	PICU stay for 1 month, discharge with normal renal function [98]
F/6	Influenza B and Enterovirus infection, post cardiac arrest	CK 23,586 IU/L	Dialysis for 1 month, normal creatinine 6 months post discharge [99]
M/12	Trauma	CK > 42,670 IU/L Myoglobin > 12,000 μg/L	Substantial drop in CK and myoglobin, discharge from PICU on Day 10 [95]
F/14	MIS-C, dystonia	CK 449,100 IU/L	Stop CKRT on Day 8 [94]
F/14	Shock, VA-ECMO with limb ischemia, anaplastic large cell lymphoma	CK 264,500 IU/L	Substantial drop in CK, succumbed 5 days after admission [98]

CK, creatine kinase; CKRT, continuous kidney replacement therapy; MIS-C, multi-system inflammatory syndrome in children, PICU, pediatric intensive care unit.

## Abbreviations

The following abbreviations are used in this manuscript:

AKI	Acute kidney injury
ATP	Adenosine triphosphate
CK	Creatine kinase
DIC	Disseminated intravascular coagulopathy
KRT	Kidney replacement therapy
